# Editorial: Human microbiome and COVID-19

**DOI:** 10.3389/fcimb.2025.1613825

**Published:** 2025-05-21

**Authors:** Mehmet Demirci, Kundlik Gadhave

**Affiliations:** ^1^ Department of Medical Microbiology, Kirklareli University School of Medicine, Kirklareli, Türkiye; ^2^ Department of Neurology, Johns Hopkins University School of Medicine, Baltimore, MD, United States; ^3^ Department of Chemistry and Biochemistry, University of Denver, Denver, CO, United States; ^4^ The Knoebel Institute for Healthy Aging, University of Denver, Denver, CO, United States; ^5^ Molecular and Cellular Biophysics Program, University of Denver, Denver, CO, United States

**Keywords:** human microbiota, COVID-19, SARS-CoV-2, pathogenesis, immune response, microbiome, pathophysiology

This Research Topic in the Frontiers in Cellular and Infection Microbiology journal highlights studies on the human microbiome and its relationship with COVID-19 pathogenesis. Although SARS-CoV-2 primarily targets the lungs in humans, it modulates the immune response in the entire host organism. The infection alters the composition of the human microbiome, both through the direct impact of COVID-19 and because of the pharmacological treatments employed during the therapeutic process (Boutin et al., 2021; Abbasi et al., 2022). The microbiome alterations include loss of beneficial commensals and an enrichment of opportunistic pathogens, linked to systemic inflammation and prolonged post-acute COVID-19 symptoms (Iqbal et al., 2025; Wang et al., 2022). This Research Topic aimed to provide a comprehensive and current overview of the intricate relationship between the human microbiome and COVID-19 pathophysiology, clinical outcomes, severity, and potential therapeutic approaches.

Liu et al.‘s study found that countries with a higher stable mortality rate (SMR) had significantly greater gut microbiome diversity compared to those with lower SMR. They identified two butyrate-producing bacteria (Eubacterium rectale and Roseburia intestinalis) that negatively correlated with SMR. They reported that lower levels of these bacteria were associated with higher COVID-19 mortality. They also indicated that the abundance of E. rectale was significantly lower in patients with severe COVID-19 compared to those with milder symptoms (Liu et al.).

Huang et al. reported that the healthy control group had a higher diversity and richness of gut bacteria than the COVID-19 group. They were found to be significantly more abundant in the COVID-19 group, including g-Streptococcus and f-Streptococcaceae, while the healthy control had higher levels of c-Clostridia and f-Ruminococcaceae. They suggested that certain gut bacteria could be potential biomarkers for COVID-19 (Huang et al.).

Fan et al. analyzed stool samples from 40 COVID-19 patients and 33 non-pneumonia controls. They found that COVID-19 patients showed significant changes in their gut microbiota, with increased opportunistic pathogens and decreased beneficial bacteria. They reported Enterococcus was more abundant in severe COVID-19 cases, while beneficial bacteria like Faecalibacterium were less common in these patients. They also reported that higher levels of Enterococcus were linked to abnormal immune cell counts in COVID-19 patients, while conversely, Faecalibacterium were positively correlated with healthy immune cell counts (Fan et al.).

In their study, Rizzello et al. analyzed stool samples from 24 hospitalized COVID-19 patients in Italy between April and May 2020 and compared them with 201 healthy individuals. They found that female COVID-19 patients had lower bacterial diversity compared to males. Both sexes showed associations with opportunistic pathogens like Enterococcus and Streptococcus, while the Candida genus was the most dominant fungal species in the gut. They reported that adult COVID-19 patients exhibited higher fungal diversity than elderly patients. They also reported that fungal Saccharomycetales were positively linked to bacterial short-chain fatty acid (SCFA) producers in the gut (Rizzello et al.).

Jiménez-Arroyo et al. included a cohort of 28 individuals diagnosed with COVID-19 in their study. They divided the individuals into two groups: those who cleared the virus within 30 days (control group), and the other were those who remained positive beyond 30 days (PCR+ group). They found that the gut microbiome of the PCR+ group showed significant differences from the control group, including higher levels of certain pathogens and increased diversity in functional gene families. They reported that an unhealthy diet was an important factor associated with prolonged viral positivity. İn the PCR+ group, higher levels of Akkermansia muciniphila, Weissella confusa, Cloacibacillus porcorum, and Parabacteroides sp. were found. Still, higher proportions of Roseburia faecis, Slackia isoflavoniconvertens, Colinsella intestinalis, and Sellimonas intestinalis were detected in the control group (Jiménez-Arroyo et al.).

Chu et al. conducted a systematic literature review by searching databases like PubMed up to August 2023. They included studies that compared microbiome data from COVID-19 and influenza patients with control groups. The 134 studies on COVID-19 and 18 studies on influenza were included. They reported that patients with COVID-19 and influenza had a lower diversity in their gut and respiratory microbiomes than healthy controls. They found that COVID-19 patients with more severe symptoms showed an even lower diversity than those with milder symptoms. They reported that harmful bacteria like Enterobacteriaceae were more abundant in COVID-19 patients, while beneficial bacteria that produce short-chain fatty acids were reduced. COVID-19 and influenza patients also showed reduced levels of beneficial bacteria like Haemophilus and Neisseria. They conclude that the microbiome of COVID-19 patients is similar to that of influenza patients, characterized by a decline in microbial diversity and specific changes in bacterial abundance (Chu et al.).

Finally, a review of Smail et al. discussed how disruptions in the gut microbiome can influence the severity and progression of COVID-19. They suggested modulating the gut microbiota could be a therapeutic strategy for COVID-19. They reported that probiotics, such as Ligilactobacillus salivarius MP101, have shown potential in reducing inflammatory mediators and improving health in at-risk individuals. They concluded that gut-derived short-chain fatty acids (SCFAs) enhance immune cell function in the lungs, while lung infections can alter gut microbiota composition (Smail et al.).

In conclusion, all studies emphasize that the human microbiome, especially the gut microbiome, is altered in COVID-19 and that its alteration may be associated with both disease and disease severity. More extensive studies are needed to understand which affects which and how fully. Understanding the coexistence and impact of SARS-CoV-2 and specific gut bacteria is crucial for clinical outcomes. It is also imperative to develop different strategies against pathogens in new outbreaks that we may encounter. We hope this Research Topic serves as a foundation for future investigations for improved microbiota-centered diagnostics and therapeutics for COVID-19 and potentially other infectious diseases.
